# Crystal structures of three salts of the tri­phenylsulfonium ion

**DOI:** 10.1107/S2056989025000118

**Published:** 2025-01-10

**Authors:** Rylan Artis, Waylan Callaway, Elizabeth Heyward, Naomi Reyes, Gavin Roberts, Kaitlyn Van Ostenbridge, Clifford W. Padgett, Will E. Lynch

**Affiliations:** a Department of Biochemistry Chemistry and Physics Georgia Southern University, 11935 Abercorn Street Savannah GA 31419 USA; Illinois State University, USA

**Keywords:** crystal structure, tri­phenyl­sulfonium ion, salts

## Abstract

The reactions of tri­phenyl­sulfonium chloride ([TPS][Cl]) with various acids in methanol yield the corresponding salts tri­phenyl­sulfonium triiodide, C_18_H_15_S^+^·I_3_^−^ or [TPS][I_3_] (**I**), tri­phenyl­sulfonium perchlorate, C_18_H_15_S^+^·ClO_4_^−^ or [TPS][ClO_4_] (**II**), and tri­phenyl­sulfonium hexa­fluoro­phosphate, C_18_H_15_S^+^·PF_6_^−^ or [TPS][PF_6_] (**III**), as crystalline products. These crystals were structurally characterized by single-crystal X-ray diffraction. In all three compounds, the sulfur atom in the tri­phenyl­sulfonium cation adopts a distorted trigonal–pyramidal geometry. [TPS][I_3_] (**I**) and [TPS][PF_6_] **(III**) both crystallize in the space group *P*2_1_/*n*, while [TPS][ClO_4_] (**II**) crystallizes in *P*2_1_. The S—C bond lengths are comparable across the three salts, and the S—C—S bond angles are consistently between 102 and 106°. Hirshfeld surface analyses reveal that each structure is dominated by hydrogen-based inter­molecular contacts, supplemented by anion-specific inter­actions such as I⋯H in (**I**), O⋯H in (**II**), and F⋯H in (**III**). These contacts organize the ions into mono-periodic ribbon- or chain-like arrangements. No significant π–π stacking is observed.

## Abstract 2

The crystal structures of three salts of tri­phenyl­sulfonium ion are reported, namely, tri­phenyl­sulfonium triiodide (**I**), tri­phenyl­sulfonium perchorate (**II**), tri­phenyl­sulfonium hexa­fluoro­phosphate (**III**).

## 1. Chemical context

Tri­phenyl­sulfonium (TPS) salts are widely used in electronic technologies, such as photoinitiators of cationic polymerizations. The basis of their activity is their direct or sensitized photolysis, which results in the release of a reactive proton and the cleavage of the C–S bond in the tri­phenyl­sulfonium cation. The process then causes solubility-changing reactions like cationic polymerization or acid-catalyzed cleavage. TPS’s ability to produce photoacids has been used to encourage desired changes in the material’s characteristics (Petsalakis *et al.*, 2014 ▸).

Tri­phenyl­sulfonium compounds are a subject of inter­est in photochemistry. More specifically, tri­phenyl­sulfonium acts as a photoacid generator meaning that it reacts and forms an acid in the presence of certain wavelengths of light (Ohmori *et al.*, 1998 ▸). This makes it useful in photolithography, ultimately also making it a subject of inter­est in the development and production of semiconductor devices or computer chips (see, for example, Kwon *et al.*, 2014 ▸ and Wang *et al.*, 2023 ▸). Additionally, tri­phenyl­sulfonium ions play a role in inhibiting mitochondrial oxidative phospho­rylation and adenosine triphosphate activity (Barrett & Selwyn, 1976 ▸), as well as in exciton emission applications in anti-counterfeiting (Luo *et al.*, 2022 ▸).

Due to a lack of readily available crystal structures of various anions complexed with tri­phenyl­sulfonium, X-ray diffraction and IR spectroscopy were used to explore the structure of multiple tri­phenyl­sulfonium cations with different anions after substitution of the chloride using the corresponding acids in excess. Herein, we report the synthesis of three complexes of the tri­phenyl­sulfonium cation (TPS^+^) with triiodide, perchlorate, and hexa­fluoro­phosphate. The complexes are formulated as [TPS][I_3_] [C_18_H_15_SI_3_, Compound (**I**)], [TPS][ClO_4_] [C_18_H_15_SClO_4_, Compound (**II**)], and [TPS][PF_6_] [C_18_H_15_SPF_6_, Compound (**III**)]. All three compounds were prepared by reacting tri­phenyl­sulfonium chloride ([TPS][Cl]) with an excess of the corresponding acid in methanol and the resulting complexes were found to have the sulfur in a trigonal–pyramidal environment.
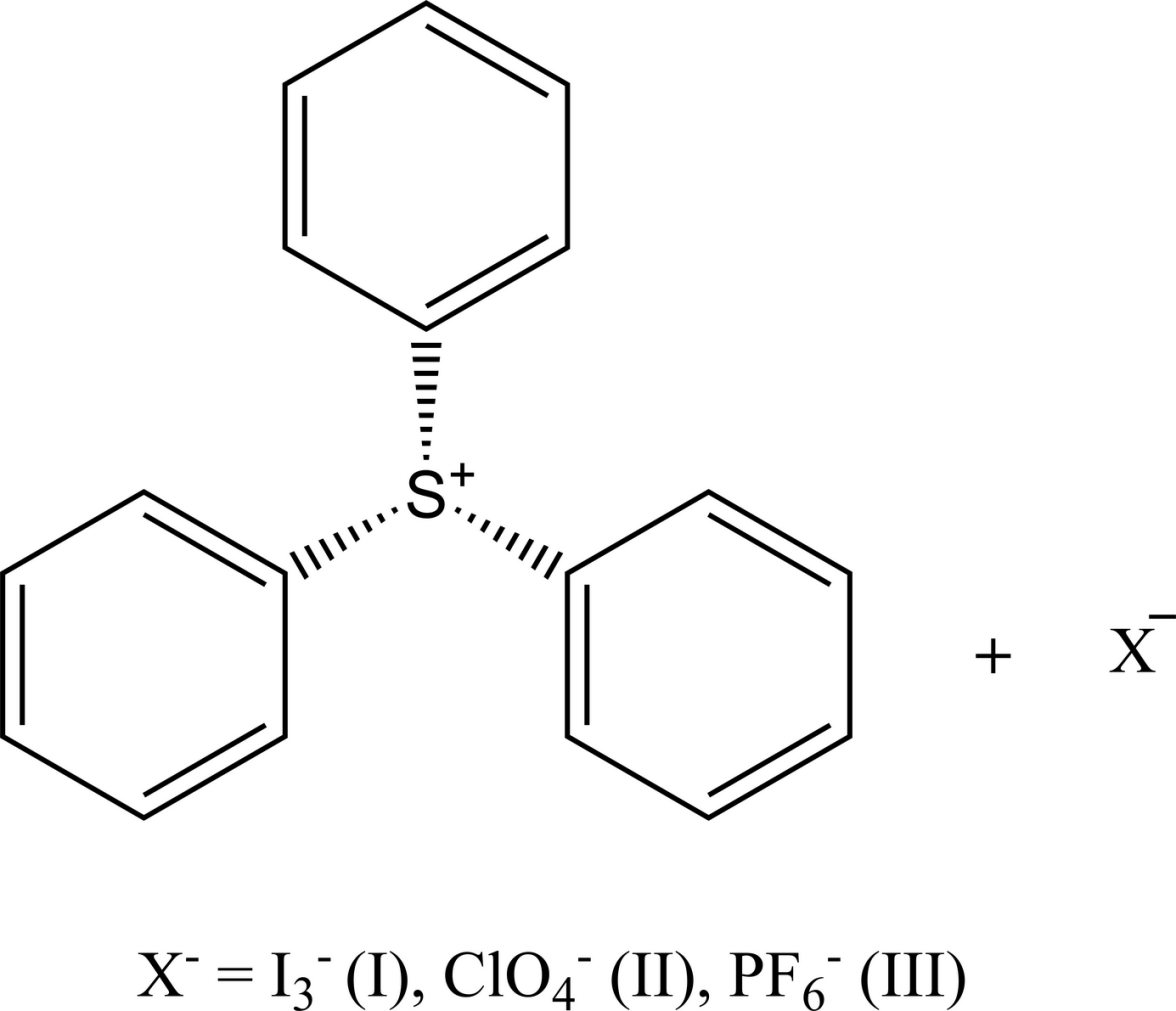


## 2. Structural commentary

**Figure 1:**
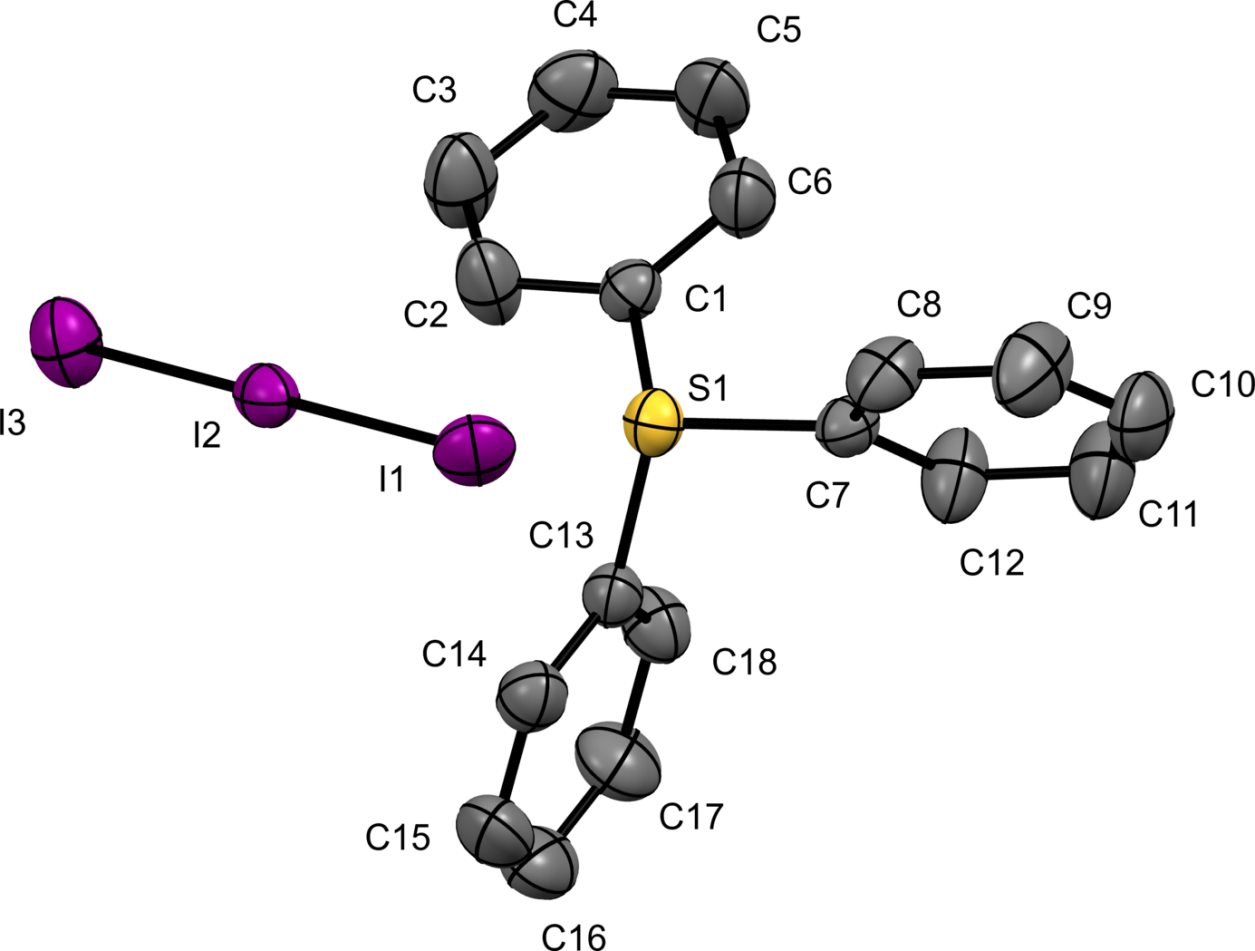
The mol­ecular structure of (**I**) with displacement ellipsoids drawn at the 50% probability level. H atoms have been omitted for clarity.

Tri­phenyl­sulfonium triiodide (**I**) crystallizes in the primitive centrosymmetric space group *P*2_1_/*n*. The asymmetric unit consists of one unit of the salt, [TPS][I_3_] (Fig. 1 ▸). The sulfur atom is observed to be in a distorted trigonal–pyramidal geometry with C1—S1—C7, C1—S1—C13, and C7—S1—C13 bond angles of 106.3 (2), 101.9 (2), and 106.2 (2)°, respectively. The sulfur atom is 3.8037 (11) Å from I2, the central iodine atom and 4.1127 (11) Å from I1, showing a close off-center contact with the triiodide anion. The sulfur–carbon bond distances are all similar, with an average of 1.787 ± 0.010 Å.

**Figure 2:**
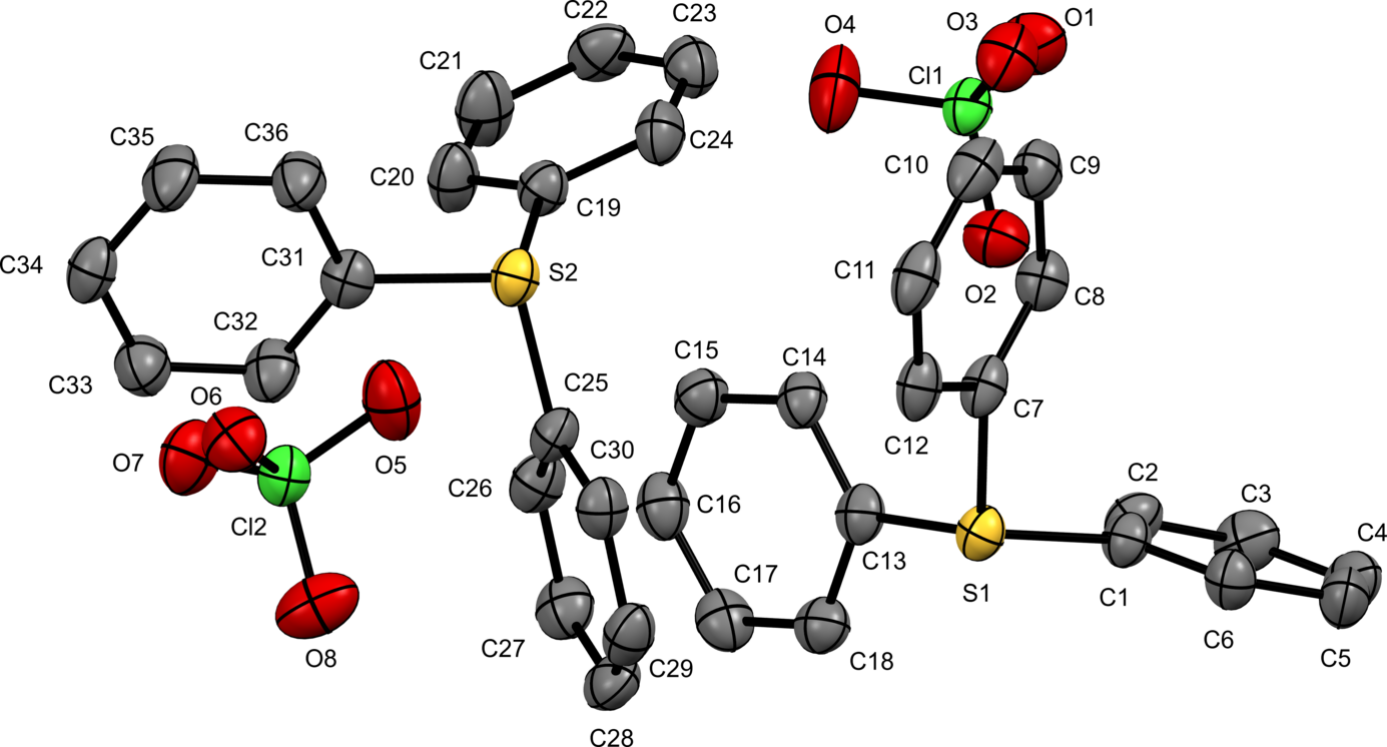
The mol­ecular structure of (**II**) with displacement ellipsoids drawn at the 50% probability level. H atoms have been omitted for clarity.

Tri­phenyl­sulfonium perchlorate (**II**) crystallizes in the space group *P*2_1_ with the asymmetric unit containing two units of the salt, [TPS][ClO_4_] (Fig. 2 ▸). Both sulfur atoms are distorted trigonal pyramidal and similar in structure to the triiodide. The C—S—C bond angles are found in the range 104.5 (3) to 106.1 (3)° and bond distances of 1.775 (6) to 1.785 (6) Å. The closest contact between the sulfur atoms and the perchlorate oxygen atoms is 3.211 (5) Å for S1⋯O6 and 3.330 (6) Å for S2⋯O4.

**Figure 3:**
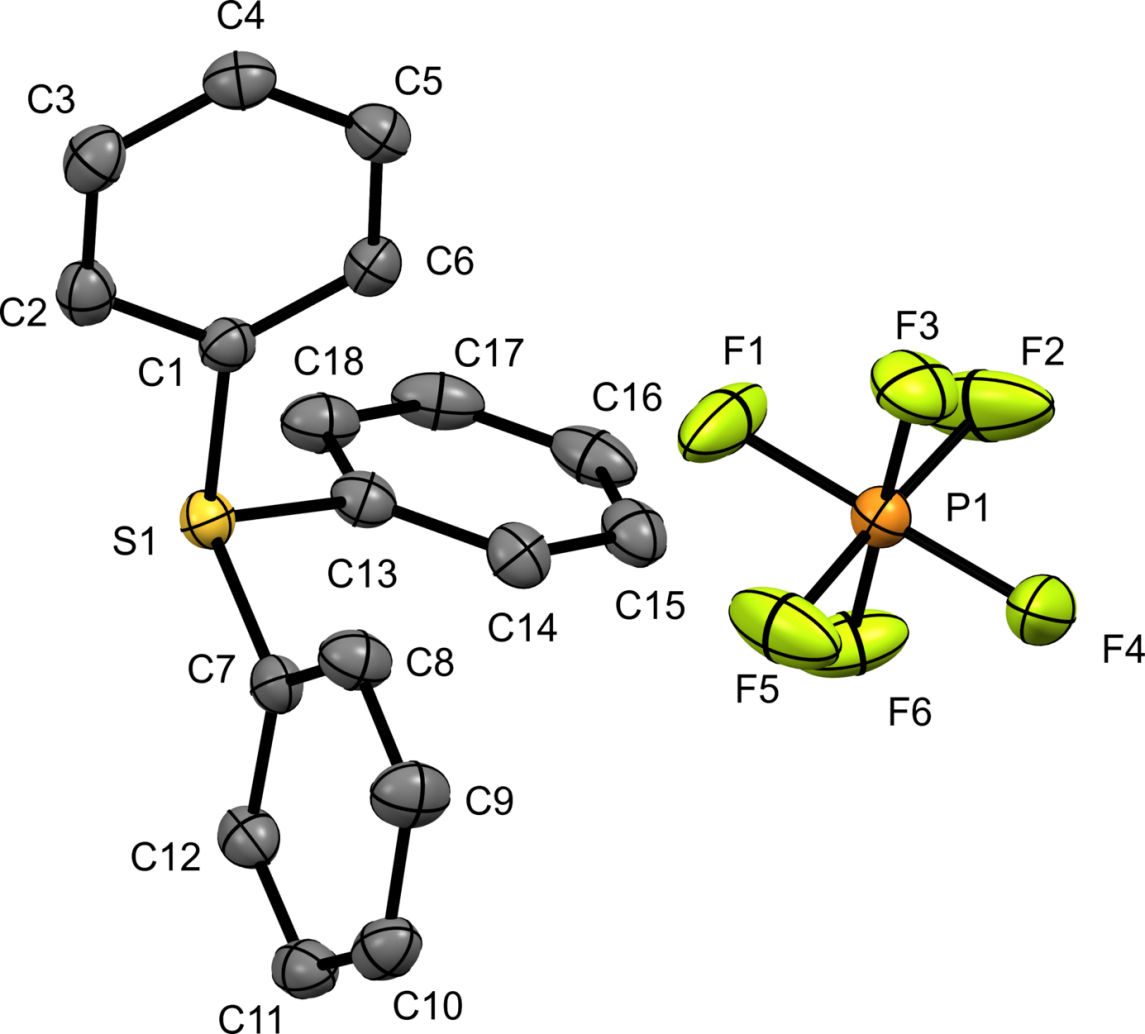
The mol­ecular structure of (**III**) with displacement ellipsoids drawn at the 50% probability level. H atoms have been omitted for clarity.

Tri­phenyl­sulfonium hexa­fluoro­phosphate (**III**), as seen in (**I**), crystallizes in the primitive centrosymmetric space group *P*2_1_/*n*. The asymmetric unit consists of one unit of the salt, [TPS][PF_6_] (Fig. 3 ▸). The sulfur atom is observed to be in a distorted trigonal–pyramidal geometry with C1—S1—C7, C1—S1—C13, and C7—S1—C13 bond angles of 105.20 (13), 104.70 (13), and 102.96 (14)°, respectively. The sulfur atom S1 is 3.287 (3) Å from the nearest fluorine atom, F2. The sulfur–carbon bond distances are all similar in the range from 1.787 (3) to 1.790 (3) Å.

In comparing the structural details of the tri­phenyl­sulfonium cation with its heavier chalcogen analogs (seleno­nium and tellurenium), the sulfonium derivative exhibits shorter bond lengths and wider C—Ch—C bond angles (Ch = Se, Te). In tri­phenyl­seleno­nium chloride hydrate (Mitcham *et al.*, 1979 ▸), the Se—C bond lengths [1.924 (4)–1.941 (4) Å] are approximately 0.15 Å longer than in the corresponding sulfonium derivative, while the C—Se—C angles [100.3 (1)–101.1 (1)°] are slightly smaller. Notable van der Waals contacts are observed for Se—Cl [3.530 (2) Å] and Se—O [3.147 (4) Å]. A similar pattern is evident in the tri­phenyl­seleno­nium chloride dihydrate dimer (Lee & Titus, 1976 ▸), with slightly longer Se—C bond distances [1.911 (10)–1.936 (12) Å] and marginally constrained C—Se—C angles [99.5 (5)–101.7 (4)°].

A more pronounced effect is observed in the tri­phenyl­tellurenium derivative, μ-(acetic acid)-di-μ-chlorido-bis­[tri­phenyl­tellurium(IV)] monohydrate (Hu *et al.*, 2013 ▸). The Te—C distances [2.116 (3)–2.129 (4) Å] are further elongated, while the C—Te—C angles [93.47 (13)–97.65 (13)°] are significantly compressed. Te—Cl close contacts [3.2007 (11) and 3.4407 (11) Å] and Te—O inter­actions [3.067 (3) and 3.113 (3) Å] are also observed. These trends reflect the larger atomic radius of the heavier chalcogens and the resulting decrease in steric hindrance. Notably, while seleno­nium and telluronium cations exhibit secondary chalcogen-bond inter­actions with Lewis-base donors, the tri­phenyl­sulfonium cation presents only van der Waals contacts, with no significant secondary S⋯*X* inter­actions evident.

## 3. Supra­molecular features

**Figure 4:**
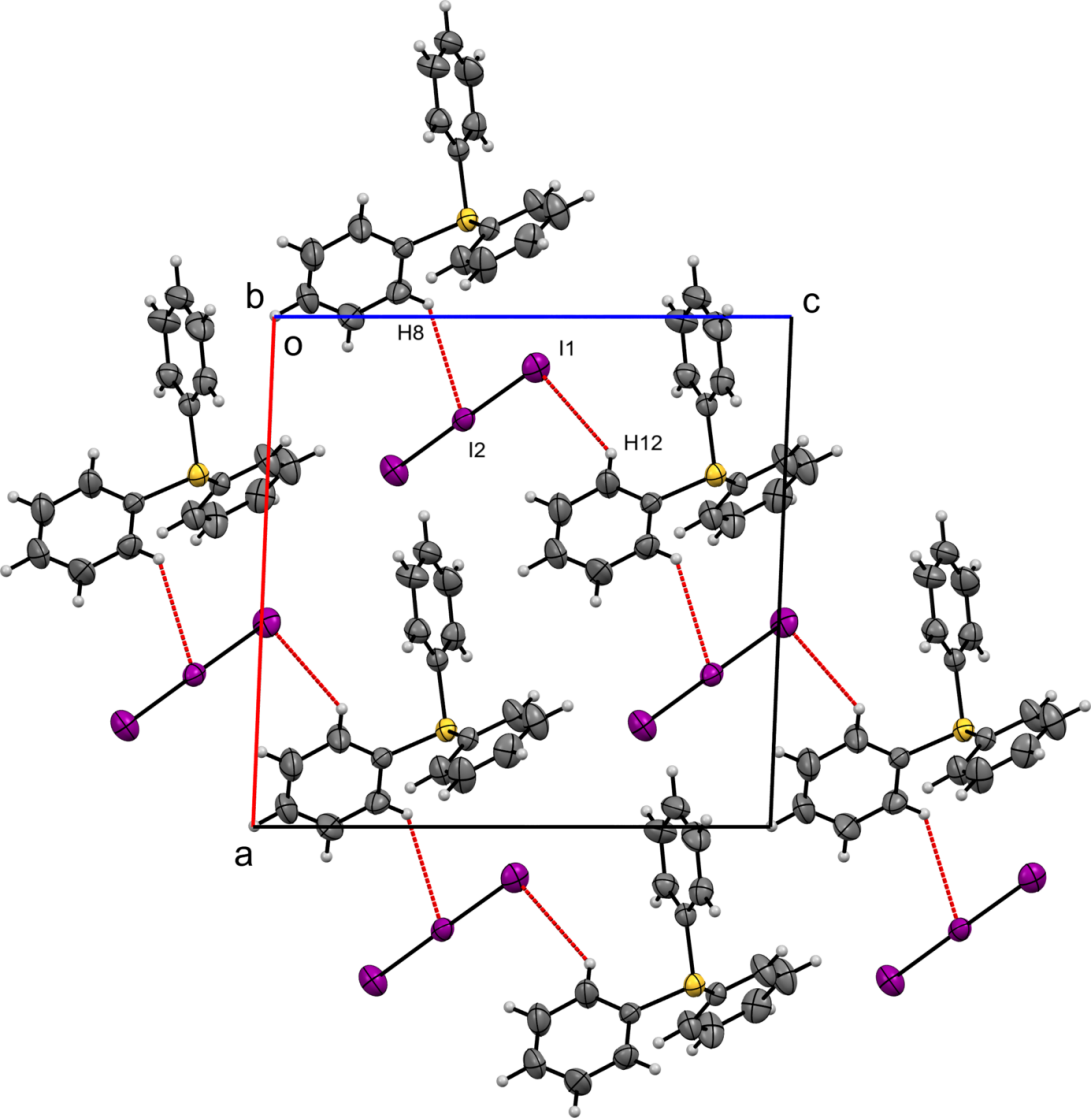
A view along the *b-*axis direction of the crystal packing of (**I**) with close contacts shown as red dashed lines.

Figs. 4 ▸, 5 ▸ and 6 ▸ show the packing of compounds (**I**), (**II**), and (**III**), respectively. In all three compounds, the packing is consolidated by van der Waals and electrostatic inter­actions, and no π–π stacking inter­actions are observed. Hirshfeld surfaces of the cations and anions were generated using *Crystal Explorer 21* (Spackman *et al.*, 2021 ▸), and the corresponding two-dimensional fingerprint plots (McKinnon *et al.*, 2007 ▸) were analyzed to qu­antify the relative contributions of the various inter­molecular contacts (Table 1 ▸).

In the crystal structure of compound (**I**), the Hirshfeld surface of the tri­phenyl­sulfonium cation is dominated by H⋯H inter­actions, which account for 46.7% of the total contacts. Significant contributions arise from H⋯C (25.1%) and H⋯I (20.5%), while C⋯C contacts are minor (3.9%). The Hirshfeld surface of the triiodide anion is strongly influenced by I⋯H contacts (84.1%), with additional contributions from I⋯I (7.1%), I⋯C (5.2%), and I⋯S (3.6%). These inter­actions result in ribbons composed of triiodide anions and tri­phenyl­sulfonium cations that extend along the [101] direction. The ribbons are concatenated by I⋯H contacts between I1 and H12 (3.134 Å) and between I2 and H8 (3.170 Å), (Fig. 4 ▸).

**Figure 5:**
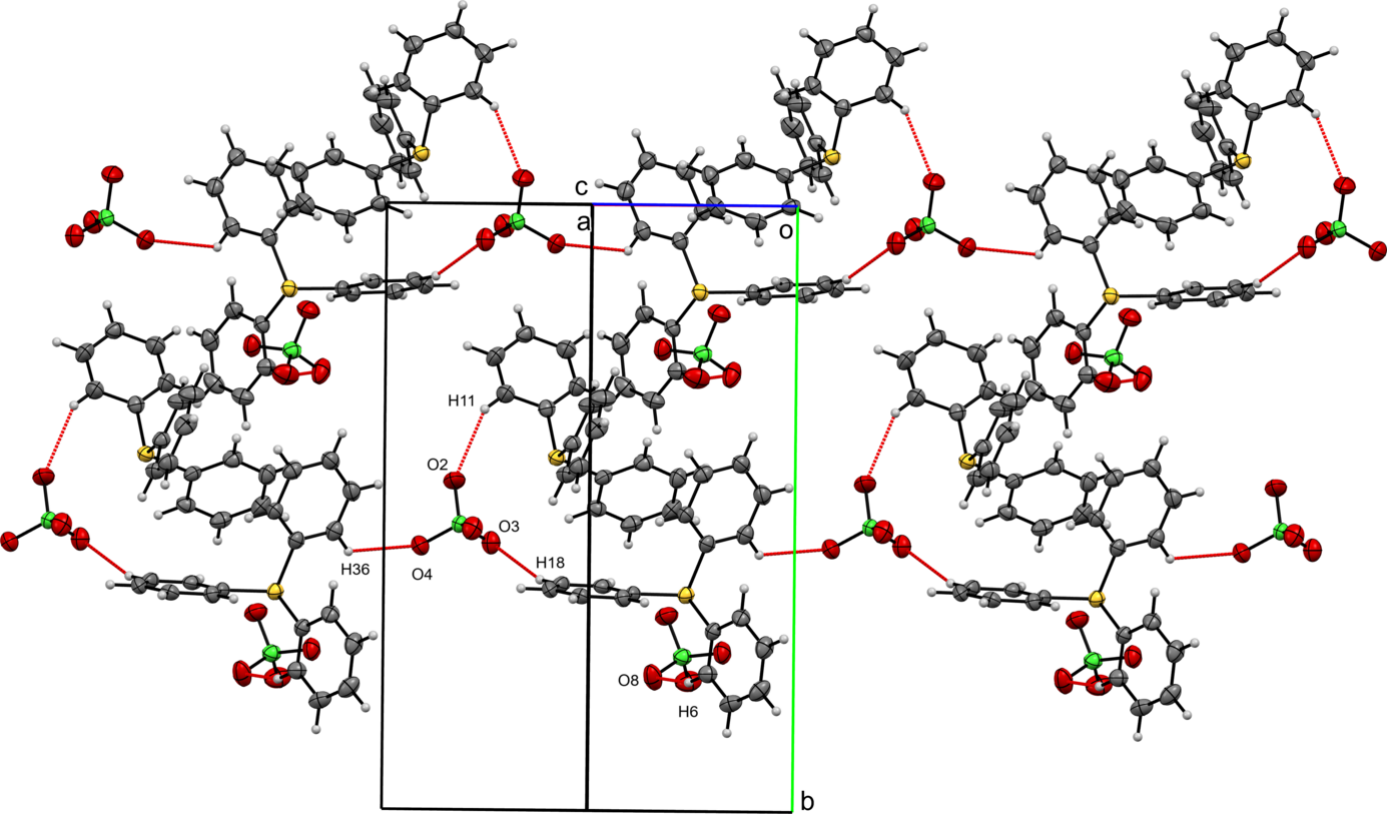
A view along the [101] direction of the crystal packing of (**II**) with close contacts shown as red dashed lines.

In the crystal structure of compound (**II**), the Hirshfeld surface of the tri­phenyl­sulfonium cation is dominated by H⋯H contacts (39.4%). Other notable inter­actions include H⋯C (30.5%) and H⋯O (25.7%), while C⋯C contacts contribute only 1.9%. For the perchlorate ion, O⋯H contacts are most significant (94.5%), with minor contributions from O⋯S (3.7%) and O⋯C (1.7%). In compound (**II**), ribbons composed of tri­phenyl­sulfonium cations and perchlorate anions zigzag along the [101] direction. These ribbons are held together by short O⋯H contacts involving phenyl hydrogen atoms of the cation and oxygen atoms of the anion. Specifically, O4⋯H36 (2.453 Å), O2⋯H11 (2.523 Å), and O3⋯H18 (2.527 Å) are shorter than the sum of the van der Waals radii for O and H (approximately 2.72 Å) (Fig. 5 ▸). A second perchlorate anion is attached to the ribbon *via* O8⋯H6 (2.548 Å), but does not directly participate in the formation of the ribbons.

**Figure 6:**
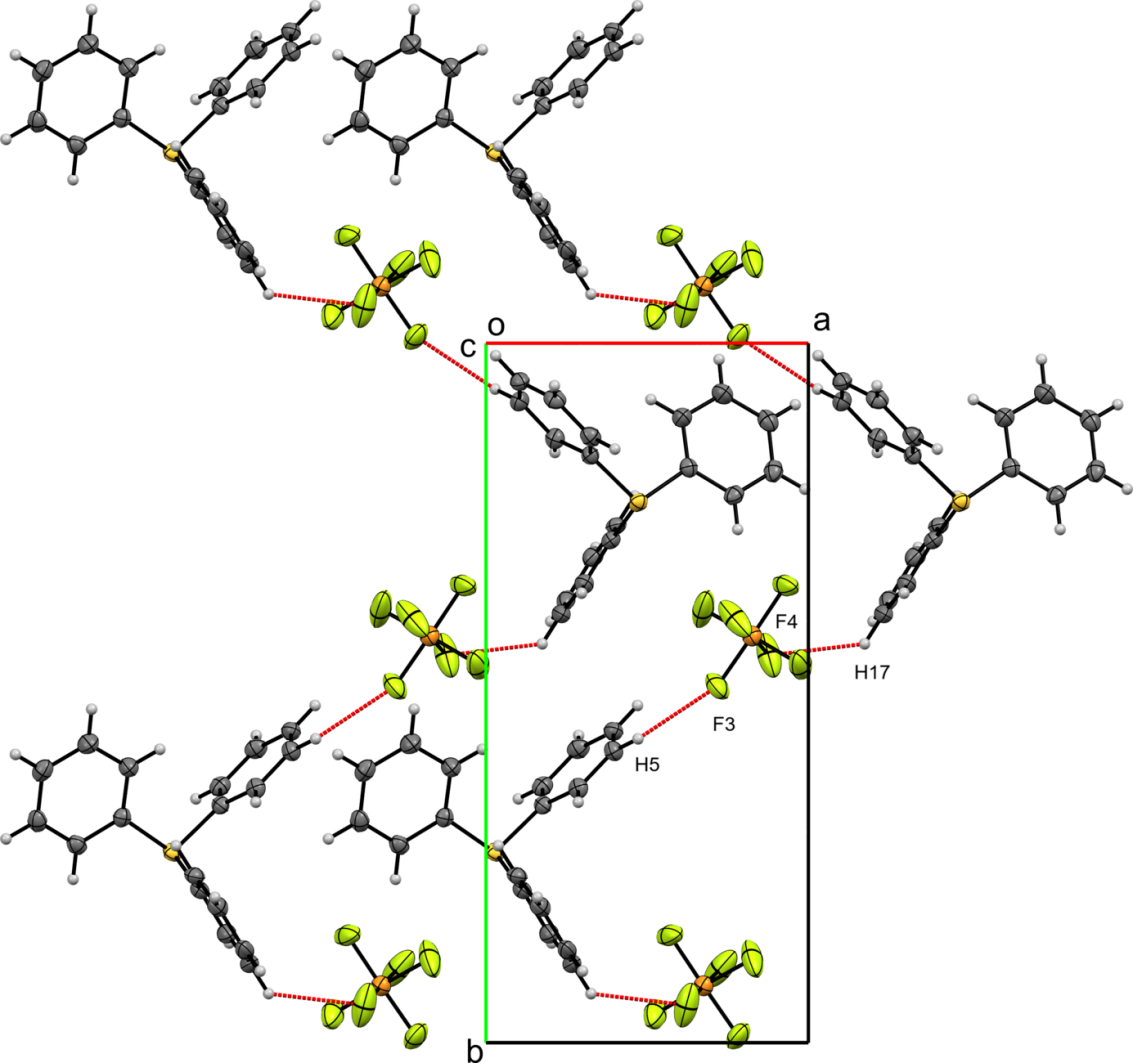
A view along the *c*-axis direction of the crystal packing of (**III**) with close contacts shown as red dashed lines.

In the crystal structure of compound (**III**), the Hirshfeld surface of the tri­phenyl­sulfonium cation is dominated by H⋯H contacts (38.9%). Other notable inter­actions include H⋯C (22.1%) and F⋯H (29.4%), while C⋯C contacts contribute only 3.7%. For the hexa­fluoro­phosphate anion, F⋯H contacts are most significant (92.4%), with smaller contributions from F⋯C (6.1%) and F⋯S (1.2%). In compound (**III**), chains of tri­phenyl­sulfonium cations and hexa­fluoro­phosphate anions zigzag along the *b*-axis direction. These chains are held together by H⋯F contacts between phenyl-ring hydrogens and anion fluorines. Specifically, F3⋯H5 (2.520 Å) and F4⋯H17 (2.510 Å) are shorter than the sum of the van der Waals radii (2.67 Å), (Fig. 6 ▸). Adjacent chains are further connected by similar H⋯F contacts, including F4⋯H3 (2.422 Å) and F1⋯H6 (2.448 Å).

## 4. Database survey

A search of the web-based Cambridge Structural Database (CSD, website, accessed on November 27, 2024; Groom *et al.*, 2016 ▸) for the tri­phenyl­sulfonium ion resulted in 18 unique entries with the majority (13) being TPS^+^ complexes. Three of the entries are nitrile or thia­zine derivatives while two are imine derivatives. The bis­[(tri­fluoro­meth­yl)sulfon­yl]aza­dine salt (BANYOH; Siu *et al.*, 2017 ▸), azide (FOYKEK; Klapötke & Krumm, 2009 ▸), tri­fluoro­methansulfonate (LECWOI; Zhang *et al.*, 2017 ▸), chloride monohydrate (NIMMIJ; Luo *et al.*, 2022 ▸), bromide hydrate (ROKYAS; Klapötke & Krumm, 2009 ▸), tetra­fluoro­borate (TUBXET; Ovchinnikov *et al.*, 1996 ▸) are aligned with this report. Transition-metal anionic salts are also reported with hexa­chloro­tin(V) (NIMMAB; Luo *et al.*, 2022 ▸), hexa­chloro­tellurium(V) (NIMMEF; Luo *et al.*, 2022 ▸), bis (μ_2_-1,3-azido)­silver(I) (QOSQEV; Klapötke *et al.*, 2009 ▸) and tris­(μ_2_-dicyanamido)­manganese(II) (SABFUX; Schlueter *et al.*, 2004 ▸).

## 5. Synthesis and crystallization

Compound (**I**) ([TPS][I_3_]) was synthesized by dissolving 0.100 g of [TPS][Cl] (0.335 mmol, purchased from TCI America) in 5 mL of methanol to which 0.500 mL of HI (57% in water, Sigma Millipore) were added. The solution was covered with parafilm then allowed to sit; X-ray quality crystals were grown by slow evaporation at room temperature. Yield, 0.0319 g (14.8%). Selected IR bands (ATR-IR, cm^−1^) : 3056 (*w*), 3021 (*w*), 1471 (*s*), 1443 (*s*), 1212 (*s*), 1143 (*s*), 1020 (*s*), 971 (*s*), 741 (*s*), 679 (*s*), 611 (*s*), 490 (*s*).

Compound (**II**) ([TPS][ClO_4_]) tri­phenyl­sulfonium perchlorate was synthesized by adding 0.500 mL of HClO_4_ (70% in water, purchased from Sigma Millipore) to 3.00 mL of 0.110 *M* [TPS][Cl] (0.330 mmol, tri­phenyl­sulfonium chloride, purchased from TCI America) methanol solution. The resulting solution was covered with a watch glass, and allowed to sit and the solvent evaporate. X-ray quality crystals were grown by slow evaporation at room temperature. Yield of [TPS][ClO_4_] 0.0842 g (70.3%). IR bands (ATR-IR, cm^−1^) : 3098 (*w*), 3027 (*w*), 1475 (*w*), 1445 (*w*), 1293 (*w*), 1076 (*s*), 996 (*w*), 745 (*m*), 680 (*m*), 622 (*s*), 504 (*m*).

Compound (**III**) ([TPS][PF_6_]) was synthesized by the addition of 0.106 g of [TPS][Cl] (0.355 mmol, purchased from TCI America) with 0.500 mL of HPF_6_ (5.65 mmol, 55% in water, purchased from Sigma Aldrich) in minimal methanol. The solution was covered with parafilm and allowed to evaporate for one week at room temperature. After vacuum filtration, the sample had a mass of 0.0677 g (46.7%). Selected IR bands from this solution (ATR-IR, cm^−1^) : 3086 (*w*), 3034 (*w*), 1475 (*s*), 1448 (*s*), 1369 (*s*), 1218 (*s*), 1055 (*s*), 993 (*s*), 858 (*s*), 850 (*s*), 827 (*s*), 745 (*s*), 680 (*s*), 555 (*s*), 496 (*s*).

## 6. Refinement

Crystal data, data collection and structure refinement details are summarized in Table 2 ▸. All carbon-bound H atoms were positioned geometrically and refined as riding: C—H = 0.95–0.98 Å with *U*_iso_(H) = 1.2*U*_eq_(C).

## Supplementary Material

Crystal structure: contains datablock(s) I, II, III. DOI: 10.1107/S2056989025000118/ej2011sup1.cif

Structure factors: contains datablock(s) I. DOI: 10.1107/S2056989025000118/ej2011Isup2.hkl

Structure factors: contains datablock(s) II. DOI: 10.1107/S2056989025000118/ej2011IIsup3.hkl

Structure factors: contains datablock(s) III. DOI: 10.1107/S2056989025000118/ej2011IIIsup4.hkl

Supporting information file. DOI: 10.1107/S2056989025000118/ej2011Isup5.cml

Supporting information file. DOI: 10.1107/S2056989025000118/ej2011IIsup6.cml

Supporting information file. DOI: 10.1107/S2056989025000118/ej2011IIIsup7.cml

CCDC references: 2414941, 2414940, 2414939

Additional supporting information:  crystallographic information; 3D view; checkCIF report

## Figures and Tables

**Table 1:** Contributions of selected inter­molecular contacts (%)

Contact	(**I**) (cation)	(**I**) (anion)	(**II**) (cation)	(**II**) (anion)	(**III**) (cation)	**III** (anion)
H⋯H	46.7	–	39.4	–	38.9	–
H⋯C	25.1	5.2	30.5	1.7	22.1	6.1
H⋯I	20.5	84.1	–	–	–	–
C⋯C	3.9	–	1.9	–	3.7	–
H⋯O	–	–	25.7	94.5	–	–
I⋯I	–	7.1	–	–	–	–
I⋯S	–	3.6	–	–	–	–
F⋯H	–	–	–	–	29.4	92.4
F⋯C	–	–	–	–	–	6.1
F⋯S	–	–	–	–	–	1.2
O⋯S	–	–	–	3.7	–	–

**Table 2:** Experimental details

	(**I**)	(**II**)	(**III**)
Crystal data			
Chemical formula	C_18_H_15_S^+^·I_3_^−^	C_18_H_15_S^+^·ClO_4_^−^	C_18_H_15_S^+^·PF_6_^−^
*M*_r_	644.06	362.81	408.33
Crystal system, space group	Monoclinic, *P*2_1_/*n*	Monoclinic, *P*2_1_	Monoclinic, *P*2_1_/*n*
Temperature (K)	299	100	100
*a*, *b*, *c* (Å)	12.8971 (1), 11.9414 (1), 13.0718 (1)	9.1289 (2), 19.1565 (4), 9.3314 (2)	8.4524 (2), 18.1483 (5), 11.4344 (3)
β (°)	92.374 (1)	90.611 (2)	98.251 (2)
*V* (Å^3^)	2011.45 (3)	1631.76 (6)	1735.84 (8)
*Z*	4	4	4
Radiation type	Cu *K*α	Cu *K*α	Cu *K*α
μ (mm^−1^)	37.53	3.45	3.10
Crystal size (mm)	0.14 × 0.10 × 0.10	0.18 × 0.17 × 0.13	0.27 × 0.18 × 0.09

Data collection			
Diffractometer	XtaLAB Synergy, Single source at home/near, HyPix3000	XtaLAB Synergy, Single source at home/near, HyPix3000	XtaLAB Synergy, Single source at home/near, HyPix3000
Absorption correction	Multi-scan (*CrysAlis PRO*; Rigaku OD, 2023 ▸)	Multi-scan (*CrysAlis PRO*; Rigaku OD, 2023 ▸)	Multi-scan (*CrysAlis PRO*; Rigaku OD, 2023 ▸)
*T*_min_, *T*_max_	0.526, 1.000	0.687, 1.000	0.225, 1.000
No. of measured, independent and observed [*I* > 2σ(*I*)] reflections	20556, 3681, 3113	14869, 5850, 5589	8136, 3235, 2782
*R*_int_	0.046	0.035	0.038
(sin θ/λ)_max_ (Å^−1^)	0.603	0.608	0.609

Refinement			
*R*[*F*^2^ > 2σ(*F*^2^)], *wR*(*F*^2^), *S*	0.031, 0.077, 1.06	0.049, 0.138, 1.07	0.057, 0.158, 1.11
No. of reflections	3681	5850	3235
No. of parameters	215	463	250
No. of restraints	0	1	0
H-atom treatment	Only H-atom displacement parameters refined	Only H-atom displacement parameters refined	Only H-atom displacement parameters refined
Δρ_max_, Δρ_min_ (e Å^−3^)	0.87, −0.90	0.59, −0.29	1.03, −0.76
Absolute structure	–	Flack *x* determined using 2436 quotients [(*I*^+^)−(*I*^−^)]/[(*I*^+^)+(*I*^−^)] (Parsons *et al.*, 2013 ▸)	–
Absolute structure parameter	–	0.005 (16)	–

Computer programs: *CrysAlis PRO* (Rigaku OD, 2023 ▸), *SHELXT2018/2* (Sheldrick, 2015*a* ▸), *SHELXL2018/3* (Sheldrick, 2015*b* ▸) and *OLEX2* (Dolomanov *et al.*, 2009 ▸).

## supplementary crystallographic information

### Triphenylsulfonium triiodide (I) Crystal data

**Table:**

C_18_H_15_S^+^·I_3_^−^	*F*(000) = 1192
*M**_r_* = 644.06	*D*_x_ = 2.127 Mg m^−^^3^
Monoclinic, *P*2_1_/*n*	Cu *K*α radiation, λ = 1.54184 Å
*a* = 12.8971 (1) Å	Cell parameters from 11125 reflections
*b* = 11.9414 (1) Å	θ = 3.4–67.9°
*c* = 13.0718 (1) Å	µ = 37.53 mm^−^^1^
β = 92.374 (1)°	*T* = 299 K
*V* = 2011.45 (3) Å^3^	Irregular, clear dark red
*Z* = 4	0.14 × 0.10 × 0.10 mm

### Triphenylsulfonium triiodide (I) Data collection

**Table:**

XtaLAB Synergy, Single source at home/near, HyPix3000 diffractometer	3113 reflections with *I* > 2σ(*I*)
Detector resolution: 10.0000 pixels mm^-1^	*R*_int_ = 0.046
ω scans	θ_max_ = 68.4°, θ_min_ = 4.7°
Absorption correction: multi-scan (CrysAlisPro; Rigaku OD, 2023)	*h* = −12→15
*T*_min_ = 0.526, *T*_max_ = 1.000	*k* = −14→14
20556 measured reflections	*l* = −15→15
3681 independent reflections	

### Triphenylsulfonium triiodide (I) Refinement

**Table:**

Refinement on *F*^2^	Hydrogen site location: inferred from neighbouring sites
Least-squares matrix: full	Only H-atom displacement parameters refined
*R*[*F*^2^ > 2σ(*F*^2^)] = 0.031	*w* = 1/[σ^2^(*F*_o_^2^) + (0.0394*P*)^2^] where *P* = (*F*_o_^2^ + 2*F*_c_^2^)/3
*wR*(*F*^2^) = 0.077	(Δ/σ)_max_ = 0.001
*S* = 1.06	Δρ_max_ = 0.87 e Å^−^^3^
3681 reflections	Δρ_min_ = −0.90 e Å^−^^3^
215 parameters	Extinction correction: *SHELXL2018/3* (Sheldrick 2015a), Fc^*^=kFc[1+0.001xFc^2^λ^3^/sin(2θ)]^-1/4^
0 restraints	Extinction coefficient: 0.00048 (3)

### Triphenylsulfonium triiodide (I) Special details

**Table:**

Geometry. All esds (except the esd in the dihedral angle between two l.s. planes) are estimated using the full covariance matrix. The cell esds are taken into account individually in the estimation of esds in distances, angles and torsion angles; correlations between esds in cell parameters are only used when they are defined by crystal symmetry. An approximate (isotropic) treatment of cell esds is used for estimating esds involving l.s. planes.

### Triphenylsulfonium triiodide (I) Fractional atomic coordinates and isotropic or equivalent isotropic displacement parameters (Å^2^)

**Table:**

	*x*	*y*	*z*	*U*_iso_*/*U*_eq_	
I1	0.09982 (3)	0.36091 (3)	0.51008 (3)	0.05011 (13)	
I2	0.20170 (2)	0.52246 (3)	0.37435 (2)	0.03829 (11)	
I3	0.30159 (3)	0.68180 (3)	0.24418 (3)	0.05604 (13)	
C1	0.1721 (3)	0.8058 (4)	0.5915 (3)	0.0330 (10)	
S1	0.18974 (8)	0.66386 (9)	0.63434 (8)	0.0331 (3)	
C2	0.2181 (4)	0.8315 (4)	0.5016 (4)	0.0536 (14)	
H2	0.256556	0.778143	0.467919	0.051 (15)*	
C3	0.2061 (5)	0.9377 (5)	0.4624 (4)	0.0636 (16)	
H3	0.236932	0.956765	0.401684	0.11 (2)*	
C4	0.1490 (4)	1.0153 (5)	0.5125 (4)	0.0596 (15)	
H4	0.142610	1.087524	0.486313	0.08 (2)*	
C5	0.1013 (5)	0.9884 (5)	0.6001 (4)	0.0625 (16)	
H5	0.062379	1.041862	0.633138	0.10 (2)*	
C6	0.1108 (4)	0.8803 (4)	0.6404 (4)	0.0507 (13)	
H6	0.076556	0.859810	0.698763	0.067 (18)*	
C7	0.1347 (3)	0.6560 (4)	0.7566 (3)	0.0339 (10)	
C8	0.0491 (4)	0.5874 (4)	0.7611 (4)	0.0460 (12)	
H8	0.025085	0.547618	0.703665	0.053 (15)*	
C9	0.0000 (4)	0.5789 (6)	0.8517 (4)	0.0659 (17)	
H9	−0.058230	0.533426	0.855703	0.09 (2)*	
C10	0.0359 (4)	0.6372 (5)	0.9367 (4)	0.0612 (16)	
H10	0.001564	0.631718	0.997675	0.060 (16)*	
C11	0.1219 (5)	0.7029 (5)	0.9316 (4)	0.0631 (16)	
H11	0.146671	0.740858	0.989704	0.08 (2)*	
C12	0.1727 (4)	0.7138 (5)	0.8415 (4)	0.0544 (14)	
H12	0.231195	0.758964	0.837948	0.062 (17)*	
C13	0.3272 (3)	0.6571 (4)	0.6570 (3)	0.0354 (10)	
C14	0.3736 (4)	0.5581 (4)	0.6286 (3)	0.0449 (12)	
H14	0.334363	0.499431	0.600619	0.038 (13)*	
C15	0.4801 (4)	0.5488 (5)	0.6430 (4)	0.0601 (15)	
H15	0.513174	0.483109	0.624400	0.064 (17)*	
C16	0.5375 (4)	0.6359 (5)	0.6848 (4)	0.0588 (15)	
H16	0.609032	0.628456	0.694308	0.055 (15)*	
C17	0.4908 (4)	0.7326 (5)	0.7122 (4)	0.0579 (15)	
H17	0.530404	0.790689	0.740656	0.11 (3)*	
C18	0.3845 (4)	0.7453 (5)	0.6979 (4)	0.0454 (12)	
H18	0.352311	0.811907	0.715467	0.068 (18)*	

### Triphenylsulfonium triiodide (I) Atomic displacement parameters (Å^2^)

**Table:**

	*U*^11^	*U*^22^	*U*^33^	*U*^12^	*U*^13^	*U*^23^
I1	0.0606 (2)	0.0358 (2)	0.0538 (2)	0.00876 (15)	0.00196 (16)	0.01070 (15)
I2	0.03714 (18)	0.0402 (2)	0.03720 (17)	0.00547 (13)	−0.00240 (13)	−0.00876 (13)
I3	0.0636 (2)	0.0514 (2)	0.0543 (2)	−0.00218 (17)	0.01768 (17)	−0.00135 (16)
C1	0.036 (2)	0.032 (2)	0.031 (2)	−0.001 (2)	−0.0007 (19)	−0.0008 (19)
S1	0.0375 (6)	0.0304 (6)	0.0314 (5)	−0.0027 (5)	0.0016 (5)	−0.0037 (4)
C2	0.071 (4)	0.042 (3)	0.050 (3)	0.009 (3)	0.025 (3)	0.003 (3)
C3	0.091 (4)	0.050 (4)	0.051 (3)	0.006 (3)	0.022 (3)	0.010 (3)
C4	0.080 (4)	0.038 (3)	0.061 (3)	0.005 (3)	−0.001 (3)	0.009 (3)
C5	0.090 (4)	0.047 (3)	0.051 (3)	0.031 (3)	0.010 (3)	−0.001 (3)
C6	0.061 (3)	0.053 (3)	0.039 (3)	0.007 (3)	0.011 (3)	0.003 (2)
C7	0.031 (2)	0.037 (3)	0.033 (2)	−0.001 (2)	−0.0011 (18)	0.003 (2)
C8	0.043 (3)	0.052 (3)	0.043 (3)	−0.014 (3)	−0.004 (2)	0.005 (2)
C9	0.052 (3)	0.092 (5)	0.054 (3)	−0.022 (3)	0.010 (3)	0.020 (3)
C10	0.062 (4)	0.085 (5)	0.038 (3)	−0.001 (3)	0.016 (3)	0.015 (3)
C11	0.075 (4)	0.079 (4)	0.037 (3)	−0.009 (4)	0.008 (3)	−0.008 (3)
C12	0.057 (3)	0.069 (4)	0.037 (3)	−0.022 (3)	0.006 (2)	−0.006 (3)
C13	0.039 (3)	0.037 (3)	0.030 (2)	0.002 (2)	0.0037 (19)	0.001 (2)
C14	0.054 (3)	0.041 (3)	0.040 (3)	0.006 (3)	0.000 (2)	0.000 (2)
C15	0.062 (4)	0.062 (4)	0.057 (3)	0.025 (3)	0.011 (3)	0.002 (3)
C16	0.037 (3)	0.082 (5)	0.059 (3)	0.005 (3)	0.009 (3)	0.012 (3)
C17	0.037 (3)	0.065 (4)	0.072 (4)	−0.004 (3)	0.009 (3)	0.000 (3)
C18	0.044 (3)	0.046 (3)	0.047 (3)	−0.002 (3)	0.006 (2)	−0.008 (2)

### Triphenylsulfonium triiodide (I) Geometric parameters (Å, º)

**Table:**

I1—I2	2.9646 (4)	C8—C9	1.370 (7)
I2—I3	2.8909 (4)	C9—H9	0.9300
C1—S1	1.797 (4)	C9—C10	1.376 (7)
C1—C2	1.373 (6)	C10—H10	0.9300
C1—C6	1.366 (6)	C10—C11	1.362 (8)
S1—C7	1.777 (5)	C11—H11	0.9300
S1—C13	1.787 (5)	C11—C12	1.378 (7)
C2—H2	0.9300	C12—H12	0.9300
C2—C3	1.373 (7)	C13—C14	1.383 (6)
C3—H3	0.9300	C13—C18	1.382 (6)
C3—C4	1.369 (7)	C14—H14	0.9300
C4—H4	0.9300	C14—C15	1.383 (7)
C4—C5	1.361 (7)	C15—H15	0.9300
C5—H5	0.9300	C15—C16	1.377 (8)
C5—C6	1.397 (7)	C16—H16	0.9300
C6—H6	0.9300	C16—C17	1.358 (8)
C7—C8	1.378 (6)	C17—H17	0.9300
C7—C12	1.380 (6)	C17—C18	1.384 (7)
C8—H8	0.9300	C18—H18	0.9300
			
I3—I2—I1	179.284 (14)	C8—C9—C10	120.6 (5)
C2—C1—S1	115.1 (4)	C10—C9—H9	119.7
C6—C1—S1	122.5 (4)	C9—C10—H10	120.0
C6—C1—C2	122.2 (5)	C11—C10—C9	119.9 (5)
C7—S1—C1	106.3 (2)	C11—C10—H10	120.0
C7—S1—C13	106.2 (2)	C10—C11—H11	119.5
C13—S1—C1	101.9 (2)	C10—C11—C12	120.9 (5)
C1—C2—H2	120.6	C12—C11—H11	119.5
C1—C2—C3	118.7 (5)	C7—C12—H12	120.8
C3—C2—H2	120.6	C11—C12—C7	118.3 (5)
C2—C3—H3	120.0	C11—C12—H12	120.8
C4—C3—C2	120.0 (5)	C14—C13—S1	115.6 (4)
C4—C3—H3	120.0	C18—C13—S1	122.7 (4)
C3—C4—H4	119.5	C18—C13—C14	121.7 (5)
C5—C4—C3	120.9 (5)	C13—C14—H14	121.0
C5—C4—H4	119.5	C15—C14—C13	118.1 (5)
C4—C5—H5	120.0	C15—C14—H14	121.0
C4—C5—C6	120.0 (5)	C14—C15—H15	119.7
C6—C5—H5	120.0	C16—C15—C14	120.5 (5)
C1—C6—C5	117.9 (5)	C16—C15—H15	119.7
C1—C6—H6	121.0	C15—C16—H16	119.7
C5—C6—H6	121.0	C17—C16—C15	120.7 (5)
C8—C7—S1	114.9 (3)	C17—C16—H16	119.7
C8—C7—C12	121.4 (4)	C16—C17—H17	119.8
C12—C7—S1	123.7 (4)	C16—C17—C18	120.4 (6)
C7—C8—H8	120.6	C18—C17—H17	119.8
C9—C8—C7	118.7 (5)	C13—C18—C17	118.6 (5)
C9—C8—H8	120.6	C13—C18—H18	120.7
C8—C9—H9	119.7	C17—C18—H18	120.7
			
C1—S1—C7—C8	114.8 (4)	C6—C1—S1—C13	−121.4 (4)
C1—S1—C7—C12	−64.8 (5)	C6—C1—C2—C3	3.3 (8)
C1—S1—C13—C14	−140.9 (3)	C7—S1—C13—C14	108.1 (4)
C1—S1—C13—C18	37.6 (4)	C7—S1—C13—C18	−73.4 (4)
C1—C2—C3—C4	−0.3 (9)	C7—C8—C9—C10	−0.5 (9)
S1—C1—C2—C3	178.5 (5)	C8—C7—C12—C11	−1.0 (8)
S1—C1—C6—C5	−179.2 (4)	C8—C9—C10—C11	−0.8 (10)
S1—C7—C8—C9	−178.1 (4)	C9—C10—C11—C12	1.3 (10)
S1—C7—C12—C11	178.5 (4)	C10—C11—C12—C7	−0.4 (9)
S1—C13—C14—C15	179.1 (4)	C12—C7—C8—C9	1.4 (8)
S1—C13—C18—C17	−179.6 (4)	C13—S1—C7—C8	−137.3 (4)
C2—C1—S1—C7	174.4 (4)	C13—S1—C7—C12	43.2 (5)
C2—C1—S1—C13	63.4 (4)	C13—C14—C15—C16	0.2 (8)
C2—C1—C6—C5	−4.4 (8)	C14—C13—C18—C17	−1.2 (7)
C2—C3—C4—C5	−1.5 (9)	C14—C15—C16—C17	−0.2 (9)
C3—C4—C5—C6	0.4 (9)	C15—C16—C17—C18	−0.4 (9)
C4—C5—C6—C1	2.5 (9)	C16—C17—C18—C13	1.1 (8)
C6—C1—S1—C7	−10.4 (4)	C18—C13—C14—C15	0.6 (7)

### Triphenylsulfonium perchlorate (II) Crystal data

**Table:**

C_18_H_15_S^+^·ClO_4_^−^	*F*(000) = 752
*M**_r_* = 362.81	*D*_x_ = 1.477 Mg m^−^^3^
Monoclinic, *P*2_1_	Cu *K*α radiation, λ = 1.54184 Å
*a* = 9.1289 (2) Å	Cell parameters from 10937 reflections
*b* = 19.1565 (4) Å	θ = 4.6–69.5°
*c* = 9.3314 (2) Å	µ = 3.45 mm^−^^1^
β = 90.611 (2)°	*T* = 100 K
*V* = 1631.76 (6) Å^3^	Block, clear colourless
*Z* = 4	0.18 × 0.17 × 0.13 mm

### Triphenylsulfonium perchlorate (II) Data collection

**Table:**

XtaLAB Synergy, Single source at home/near, HyPix3000 diffractometer	5589 reflections with *I* > 2σ(*I*)
Detector resolution: 10.0000 pixels mm^-1^	*R*_int_ = 0.035
ω scans	θ_max_ = 69.6°, θ_min_ = 4.6°
Absorption correction: multi-scan (CrysAlisPro; Rigaku OD, 2023)	*h* = −11→8
*T*_min_ = 0.687, *T*_max_ = 1.000	*k* = −23→23
14869 measured reflections	*l* = −11→11
5850 independent reflections	

### Triphenylsulfonium perchlorate (II) Refinement

**Table:**

Refinement on *F*^2^	Hydrogen site location: inferred from neighbouring sites
Least-squares matrix: full	Only H-atom displacement parameters refined
*R*[*F*^2^ > 2σ(*F*^2^)] = 0.049	*w* = 1/[σ^2^(*F*_o_^2^) + (0.0965*P*)^2^ + 0.5199*P*] where *P* = (*F*_o_^2^ + 2*F*_c_^2^)/3
*wR*(*F*^2^) = 0.138	(Δ/σ)_max_ < 0.001
*S* = 1.07	Δρ_max_ = 0.59 e Å^−^^3^
5850 reflections	Δρ_min_ = −0.29 e Å^−^^3^
463 parameters	Absolute structure: Flack *x* determined using 2436 quotients [(*I*^+^)-(*I*^-^)]/[(*I*^+^)+(*I*^-^)] (Parsons *et al.*, 2013)
1 restraint	Absolute structure parameter: 0.005 (16)

### Triphenylsulfonium perchlorate (II) Special details

**Table:**

Geometry. All esds (except the esd in the dihedral angle between two l.s. planes) are estimated using the full covariance matrix. The cell esds are taken into account individually in the estimation of esds in distances, angles and torsion angles; correlations between esds in cell parameters are only used when they are defined by crystal symmetry. An approximate (isotropic) treatment of cell esds is used for estimating esds involving l.s. planes.

### Triphenylsulfonium perchlorate (II) Fractional atomic coordinates and isotropic or equivalent isotropic displacement parameters (Å^2^)

**Table:**

	*x*	*y*	*z*	*U*_iso_*/*U*_eq_	
C1	0.2044 (6)	0.7027 (3)	0.1890 (6)	0.0318 (11)	
S1	0.35386 (13)	0.64327 (7)	0.17331 (14)	0.0303 (3)	
C2	0.0596 (6)	0.6800 (3)	0.1973 (6)	0.0358 (12)	
H2	0.037533	0.631771	0.206032	0.034 (17)*	
C3	−0.0510 (7)	0.7290 (4)	0.1926 (7)	0.0432 (15)	
H3	−0.150320	0.714586	0.197767	0.05 (2)*	
C4	−0.0169 (7)	0.8001 (4)	0.1802 (7)	0.0441 (15)	
H4	−0.093435	0.833623	0.174811	0.024 (14)*	
C5	0.1280 (7)	0.8218 (3)	0.1757 (7)	0.0426 (14)	
H5	0.150190	0.870178	0.171440	0.11 (4)*	
C6	0.2404 (6)	0.7732 (3)	0.1775 (6)	0.0350 (12)	
H6	0.339741	0.787635	0.171093	0.028 (15)*	
C7	0.4387 (5)	0.6418 (3)	0.3457 (6)	0.0316 (11)	
C8	0.3620 (6)	0.6493 (3)	0.4708 (6)	0.0341 (11)	
H8	0.258956	0.656318	0.469024	0.036 (17)*	
C9	0.4391 (7)	0.6463 (3)	0.5996 (7)	0.0367 (12)	
H9	0.389008	0.651335	0.687737	0.031 (16)*	
C10	0.5901 (7)	0.6359 (3)	0.5995 (7)	0.0408 (14)	
H10	0.642890	0.634564	0.687798	0.046 (19)*	
C11	0.6642 (6)	0.6274 (3)	0.4714 (7)	0.0404 (14)	
H11	0.767026	0.619718	0.472529	0.08 (3)*	
C12	0.5897 (6)	0.6299 (3)	0.3438 (7)	0.0355 (12)	
H12	0.639415	0.623770	0.255745	0.017 (13)*	
C13	0.2743 (6)	0.5587 (3)	0.1525 (6)	0.0332 (11)	
C14	0.2696 (6)	0.5106 (3)	0.2626 (6)	0.0335 (12)	
H14	0.301693	0.522652	0.356603	0.021 (13)*	
C15	0.2162 (6)	0.4436 (3)	0.2323 (7)	0.0379 (12)	
H15	0.213488	0.409580	0.306196	0.045 (19)*	
C16	0.1676 (6)	0.4266 (3)	0.0963 (7)	0.0402 (13)	
H16	0.131002	0.381074	0.076737	0.041 (19)*	
C17	0.1724 (7)	0.4766 (3)	−0.0126 (7)	0.0413 (13)	
H17	0.137137	0.465164	−0.105869	0.06 (2)*	
C18	0.2278 (6)	0.5424 (3)	0.0141 (6)	0.0367 (12)	
H18	0.234054	0.575859	−0.060609	0.05 (2)*	
S2	0.78712 (14)	0.41815 (7)	0.37637 (15)	0.0317 (3)	
C19	0.6226 (6)	0.3956 (3)	0.4650 (6)	0.0327 (11)	
C20	0.5543 (7)	0.3316 (3)	0.4456 (8)	0.0437 (14)	
H20	0.595442	0.297114	0.384986	0.040 (18)*	
C21	0.4251 (8)	0.3194 (4)	0.5164 (9)	0.0513 (17)	
H21	0.376742	0.275797	0.504834	0.044 (19)*	
C22	0.3651 (7)	0.3696 (4)	0.6037 (7)	0.0454 (15)	
H22	0.276868	0.360253	0.653183	0.045 (19)*	
C23	0.4334 (7)	0.4338 (3)	0.6195 (7)	0.0422 (14)	
H23	0.390092	0.468996	0.676836	0.029 (15)*	
C24	0.5640 (7)	0.4464 (3)	0.5521 (7)	0.0396 (13)	
H24	0.613222	0.489675	0.565377	0.06 (2)*	
C25	0.7284 (6)	0.4499 (3)	0.2065 (6)	0.0318 (11)	
C26	0.6048 (6)	0.4252 (3)	0.1354 (7)	0.0375 (12)	
H26	0.543828	0.391264	0.179111	0.032 (16)*	
C27	0.5714 (7)	0.4504 (3)	0.0004 (7)	0.0414 (13)	
H27	0.488593	0.433205	−0.051068	0.05 (2)*	
C28	0.6610 (7)	0.5016 (3)	−0.0595 (7)	0.0419 (14)	
H28	0.636577	0.520018	−0.151155	0.045 (19)*	
C29	0.7842 (6)	0.5260 (3)	0.0117 (7)	0.0403 (13)	
H29	0.845196	0.559702	−0.032590	0.07 (3)*	
C30	0.8191 (6)	0.5013 (3)	0.1479 (6)	0.0353 (12)	
H30	0.901706	0.518603	0.199483	0.017 (13)*	
C31	0.8756 (6)	0.3373 (3)	0.3387 (6)	0.0323 (11)	
C32	0.8534 (7)	0.3007 (3)	0.2141 (7)	0.0415 (13)	
H32	0.786457	0.317396	0.143327	0.10 (4)*	
C33	0.9296 (7)	0.2393 (4)	0.1928 (7)	0.0436 (14)	
H33	0.915223	0.213784	0.106540	0.07 (3)*	
C34	1.0262 (6)	0.2148 (3)	0.2953 (7)	0.0386 (13)	
H34	1.078994	0.172767	0.280070	0.014 (12)*	
C35	1.0453 (8)	0.2522 (4)	0.4203 (8)	0.0494 (16)	
H35	1.110788	0.235042	0.491891	0.13 (5)*	
C36	0.9717 (7)	0.3136 (3)	0.4436 (7)	0.0439 (14)	
H36	0.986355	0.339286	0.529737	0.08 (3)*	
Cl2	0.40399 (14)	0.24527 (7)	0.05278 (15)	0.0356 (3)	
O5	0.3284 (5)	0.2851 (3)	0.1575 (6)	0.0570 (13)	
O6	0.5553 (5)	0.2378 (3)	0.0957 (5)	0.0460 (10)	
O7	0.3381 (6)	0.1780 (3)	0.0402 (6)	0.0522 (12)	
O8	0.3976 (5)	0.2790 (3)	−0.0831 (6)	0.0555 (13)	
Cl1	0.02629 (13)	0.52914 (6)	0.60686 (14)	0.0322 (3)	
O1	−0.1090 (5)	0.5360 (3)	0.6796 (5)	0.0478 (11)	
O2	0.0126 (5)	0.5594 (3)	0.4671 (5)	0.0440 (10)	
O3	0.1399 (5)	0.5650 (2)	0.6847 (5)	0.0460 (11)	
O4	0.0659 (6)	0.4575 (3)	0.5948 (7)	0.0602 (14)	

### Triphenylsulfonium perchlorate (II) Atomic displacement parameters (Å^2^)

**Table:**

	*U*^11^	*U*^22^	*U*^33^	*U*^12^	*U*^13^	*U*^23^
C1	0.027 (3)	0.032 (3)	0.036 (3)	0.004 (2)	−0.002 (2)	0.001 (2)
S1	0.0237 (5)	0.0300 (6)	0.0372 (6)	0.0014 (5)	0.0000 (5)	0.0019 (5)
C2	0.032 (3)	0.035 (3)	0.041 (3)	−0.005 (2)	0.000 (2)	0.007 (2)
C3	0.027 (3)	0.054 (4)	0.048 (3)	0.001 (3)	0.001 (2)	0.007 (3)
C4	0.035 (3)	0.049 (4)	0.048 (3)	0.014 (3)	0.009 (3)	0.010 (3)
C5	0.050 (4)	0.031 (3)	0.047 (3)	0.008 (3)	0.006 (3)	0.002 (2)
C6	0.029 (3)	0.032 (3)	0.044 (3)	0.000 (2)	0.004 (2)	0.000 (2)
C7	0.024 (2)	0.024 (2)	0.047 (3)	0.000 (2)	−0.009 (2)	0.000 (2)
C8	0.028 (3)	0.031 (3)	0.044 (3)	−0.001 (2)	−0.004 (2)	−0.001 (2)
C9	0.041 (3)	0.027 (3)	0.043 (3)	−0.001 (2)	−0.005 (2)	−0.005 (2)
C10	0.041 (3)	0.029 (3)	0.052 (4)	−0.005 (2)	−0.020 (3)	0.002 (2)
C11	0.029 (3)	0.027 (3)	0.065 (4)	0.002 (2)	−0.013 (3)	0.001 (3)
C12	0.028 (3)	0.022 (3)	0.056 (3)	−0.002 (2)	−0.001 (2)	−0.001 (2)
C13	0.024 (2)	0.033 (3)	0.043 (3)	0.005 (2)	−0.002 (2)	−0.001 (2)
C14	0.030 (3)	0.032 (3)	0.038 (3)	0.005 (2)	−0.005 (2)	−0.002 (2)
C15	0.036 (3)	0.037 (3)	0.041 (3)	0.004 (2)	0.004 (2)	0.003 (2)
C16	0.033 (3)	0.037 (3)	0.051 (4)	0.003 (2)	−0.004 (2)	−0.008 (3)
C17	0.036 (3)	0.044 (3)	0.044 (3)	0.002 (3)	−0.007 (2)	−0.008 (3)
C18	0.032 (3)	0.041 (3)	0.038 (3)	0.005 (2)	−0.001 (2)	0.002 (2)
S2	0.0284 (6)	0.0271 (6)	0.0396 (7)	−0.0009 (5)	−0.0013 (5)	0.0000 (5)
C19	0.029 (3)	0.032 (3)	0.037 (3)	0.002 (2)	0.001 (2)	0.002 (2)
C20	0.044 (3)	0.027 (3)	0.061 (4)	−0.002 (2)	0.011 (3)	0.000 (3)
C21	0.048 (4)	0.034 (3)	0.072 (5)	−0.006 (3)	0.015 (3)	0.001 (3)
C22	0.038 (3)	0.050 (4)	0.048 (4)	0.001 (3)	0.012 (3)	0.014 (3)
C23	0.041 (3)	0.042 (3)	0.044 (3)	0.008 (3)	0.000 (3)	−0.004 (3)
C24	0.042 (3)	0.029 (3)	0.048 (3)	0.002 (2)	−0.004 (3)	−0.006 (2)
C25	0.030 (3)	0.026 (3)	0.040 (3)	0.004 (2)	0.001 (2)	0.006 (2)
C26	0.032 (3)	0.034 (3)	0.047 (3)	−0.002 (2)	0.003 (2)	0.006 (2)
C27	0.032 (3)	0.045 (3)	0.047 (3)	0.006 (2)	−0.006 (2)	−0.001 (3)
C28	0.042 (3)	0.042 (3)	0.042 (3)	0.011 (3)	−0.002 (2)	0.010 (3)
C29	0.038 (3)	0.031 (3)	0.052 (3)	0.002 (2)	0.009 (3)	0.008 (3)
C30	0.030 (3)	0.030 (3)	0.045 (3)	−0.003 (2)	0.003 (2)	−0.002 (2)
C31	0.023 (2)	0.033 (3)	0.041 (3)	−0.002 (2)	0.001 (2)	0.001 (2)
C32	0.036 (3)	0.040 (3)	0.048 (3)	0.007 (2)	−0.011 (3)	−0.002 (3)
C33	0.049 (3)	0.039 (3)	0.043 (3)	0.004 (3)	−0.005 (3)	−0.006 (3)
C34	0.029 (3)	0.031 (3)	0.055 (4)	0.004 (2)	0.000 (2)	0.004 (3)
C35	0.050 (4)	0.042 (4)	0.056 (4)	0.014 (3)	−0.017 (3)	0.002 (3)
C36	0.052 (4)	0.037 (3)	0.042 (3)	0.007 (3)	−0.011 (3)	−0.006 (3)
Cl2	0.0308 (6)	0.0316 (6)	0.0443 (7)	−0.0009 (5)	0.0010 (5)	0.0001 (5)
O5	0.049 (3)	0.048 (3)	0.074 (4)	0.004 (2)	0.015 (2)	−0.011 (2)
O6	0.033 (2)	0.055 (3)	0.050 (3)	0.0039 (19)	−0.0076 (18)	−0.002 (2)
O7	0.053 (3)	0.035 (2)	0.069 (3)	−0.010 (2)	−0.003 (2)	0.000 (2)
O8	0.035 (2)	0.071 (3)	0.060 (3)	−0.008 (2)	−0.008 (2)	0.023 (3)
Cl1	0.0268 (6)	0.0307 (6)	0.0390 (6)	0.0024 (5)	−0.0048 (5)	−0.0006 (5)
O1	0.036 (2)	0.057 (3)	0.050 (2)	−0.001 (2)	0.0046 (18)	0.006 (2)
O2	0.037 (2)	0.055 (3)	0.040 (2)	0.0002 (19)	−0.0010 (17)	−0.0004 (19)
O3	0.041 (2)	0.040 (2)	0.057 (3)	−0.0052 (18)	−0.013 (2)	−0.002 (2)
O4	0.058 (3)	0.033 (2)	0.090 (4)	0.012 (2)	−0.022 (3)	−0.011 (2)

### Triphenylsulfonium perchlorate (II) Geometric parameters (Å, º)

**Table:**

C1—S1	1.784 (6)	C19—C24	1.380 (8)
C1—C2	1.394 (8)	C20—H20	0.9500
C1—C6	1.396 (8)	C20—C21	1.378 (9)
S1—C7	1.778 (6)	C21—H21	0.9500
S1—C13	1.785 (6)	C21—C22	1.378 (10)
C2—H2	0.9500	C22—H22	0.9500
C2—C3	1.378 (9)	C22—C23	1.385 (10)
C3—H3	0.9500	C23—H23	0.9500
C3—C4	1.402 (10)	C23—C24	1.375 (9)
C4—H4	0.9500	C24—H24	0.9500
C4—C5	1.387 (9)	C25—C26	1.386 (8)
C5—H5	0.9500	C25—C30	1.401 (8)
C5—C6	1.385 (8)	C26—H26	0.9500
C6—H6	0.9500	C26—C27	1.381 (9)
C7—C8	1.375 (8)	C27—H27	0.9500
C7—C12	1.397 (8)	C27—C28	1.397 (10)
C8—H8	0.9500	C28—H28	0.9500
C8—C9	1.388 (8)	C28—C29	1.382 (9)
C9—H9	0.9500	C29—H29	0.9500
C9—C10	1.393 (9)	C29—C30	1.390 (9)
C10—H10	0.9500	C30—H30	0.9500
C10—C11	1.390 (10)	C31—C32	1.370 (9)
C11—H11	0.9500	C31—C36	1.383 (9)
C11—C12	1.366 (9)	C32—H32	0.9500
C12—H12	0.9500	C32—C33	1.382 (9)
C13—C14	1.382 (8)	C33—H33	0.9500
C13—C18	1.390 (8)	C33—C34	1.377 (9)
C14—H14	0.9500	C34—H34	0.9500
C14—C15	1.400 (9)	C34—C35	1.378 (10)
C15—H15	0.9500	C35—H35	0.9500
C15—C16	1.380 (9)	C35—C36	1.375 (9)
C16—H16	0.9500	C36—H36	0.9500
C16—C17	1.397 (10)	Cl2—O5	1.424 (5)
C17—H17	0.9500	Cl2—O6	1.441 (4)
C17—C18	1.380 (9)	Cl2—O7	1.427 (5)
C18—H18	0.9500	Cl2—O8	1.424 (5)
S2—C19	1.776 (6)	Cl1—O1	1.422 (5)
S2—C25	1.776 (6)	Cl1—O2	1.432 (5)
S2—C31	1.784 (6)	Cl1—O3	1.434 (4)
C19—C20	1.387 (8)	Cl1—O4	1.424 (5)
			
C2—C1—S1	122.2 (4)	C19—C20—H20	120.9
C2—C1—C6	122.0 (5)	C21—C20—C19	118.3 (6)
C6—C1—S1	115.5 (4)	C21—C20—H20	120.9
C1—S1—C13	106.1 (3)	C20—C21—H21	119.6
C7—S1—C1	105.2 (3)	C20—C21—C22	120.8 (6)
C7—S1—C13	104.9 (3)	C22—C21—H21	119.6
C1—C2—H2	120.6	C21—C22—H22	120.0
C3—C2—C1	118.8 (6)	C21—C22—C23	120.1 (6)
C3—C2—H2	120.6	C23—C22—H22	120.0
C2—C3—H3	120.0	C22—C23—H23	120.0
C2—C3—C4	120.1 (6)	C24—C23—C22	120.0 (6)
C4—C3—H3	120.0	C24—C23—H23	120.0
C3—C4—H4	119.8	C19—C24—H24	120.4
C5—C4—C3	120.4 (6)	C23—C24—C19	119.2 (6)
C5—C4—H4	119.8	C23—C24—H24	120.4
C4—C5—H5	119.8	C26—C25—S2	123.1 (4)
C6—C5—C4	120.4 (6)	C26—C25—C30	122.4 (5)
C6—C5—H5	119.8	C30—C25—S2	114.5 (4)
C1—C6—H6	120.8	C25—C26—H26	120.4
C5—C6—C1	118.4 (5)	C27—C26—C25	119.2 (5)
C5—C6—H6	120.8	C27—C26—H26	120.4
C8—C7—S1	123.0 (4)	C26—C27—H27	120.5
C8—C7—C12	122.6 (5)	C26—C27—C28	119.1 (6)
C12—C7—S1	114.3 (5)	C28—C27—H27	120.5
C7—C8—H8	120.9	C27—C28—H28	119.2
C7—C8—C9	118.2 (5)	C29—C28—C27	121.5 (6)
C9—C8—H8	120.9	C29—C28—H28	119.2
C8—C9—H9	120.1	C28—C29—H29	120.0
C8—C9—C10	119.9 (6)	C28—C29—C30	120.1 (5)
C10—C9—H9	120.1	C30—C29—H29	120.0
C9—C10—H10	119.7	C25—C30—H30	121.1
C11—C10—C9	120.5 (5)	C29—C30—C25	117.7 (5)
C11—C10—H10	119.7	C29—C30—H30	121.1
C10—C11—H11	119.9	C32—C31—S2	123.2 (5)
C12—C11—C10	120.2 (5)	C32—C31—C36	121.4 (6)
C12—C11—H11	119.9	C36—C31—S2	115.4 (5)
C7—C12—H12	120.8	C31—C32—H32	120.5
C11—C12—C7	118.5 (6)	C31—C32—C33	119.1 (6)
C11—C12—H12	120.8	C33—C32—H32	120.5
C14—C13—S1	122.7 (4)	C32—C33—H33	119.7
C14—C13—C18	121.9 (6)	C34—C33—C32	120.7 (6)
C18—C13—S1	115.2 (5)	C34—C33—H33	119.7
C13—C14—H14	120.8	C33—C34—H34	120.5
C13—C14—C15	118.3 (5)	C33—C34—C35	119.0 (6)
C15—C14—H14	120.8	C35—C34—H34	120.5
C14—C15—H15	119.6	C34—C35—H35	119.3
C16—C15—C14	120.8 (6)	C36—C35—C34	121.4 (6)
C16—C15—H15	119.6	C36—C35—H35	119.3
C15—C16—H16	120.2	C31—C36—H36	120.8
C15—C16—C17	119.6 (6)	C35—C36—C31	118.4 (6)
C17—C16—H16	120.2	C35—C36—H36	120.8
C16—C17—H17	119.7	O5—Cl2—O6	109.4 (3)
C18—C17—C16	120.6 (6)	O5—Cl2—O7	109.5 (3)
C18—C17—H17	119.7	O5—Cl2—O8	110.7 (4)
C13—C18—H18	120.6	O7—Cl2—O6	109.6 (3)
C17—C18—C13	118.8 (6)	O8—Cl2—O6	108.8 (3)
C17—C18—H18	120.6	O8—Cl2—O7	108.8 (3)
C19—S2—C31	105.5 (3)	O1—Cl1—O2	109.2 (3)
C25—S2—C19	104.5 (3)	O1—Cl1—O3	109.9 (3)
C25—S2—C31	104.8 (3)	O1—Cl1—O4	110.5 (3)
C20—C19—S2	122.4 (5)	O2—Cl1—O3	108.8 (3)
C24—C19—S2	116.0 (5)	O4—Cl1—O2	109.8 (3)
C24—C19—C20	121.6 (6)	O4—Cl1—O3	108.5 (3)
			
C1—S1—C7—C8	33.3 (5)	S2—C19—C20—C21	−178.5 (6)
C1—S1—C7—C12	−148.8 (4)	S2—C19—C24—C23	177.3 (5)
C1—S1—C13—C14	−103.2 (5)	S2—C25—C26—C27	−176.8 (5)
C1—S1—C13—C18	81.8 (5)	S2—C25—C30—C29	176.7 (4)
C1—C2—C3—C4	−0.2 (10)	S2—C31—C32—C33	178.4 (5)
S1—C1—C2—C3	−172.6 (5)	S2—C31—C36—C35	−179.0 (5)
S1—C1—C6—C5	174.3 (5)	C19—S2—C25—C26	−32.3 (5)
S1—C7—C8—C9	179.1 (4)	C19—S2—C25—C30	149.0 (4)
S1—C7—C12—C11	−179.5 (4)	C19—S2—C31—C32	90.4 (6)
S1—C13—C14—C15	−174.6 (4)	C19—S2—C31—C36	−90.6 (5)
S1—C13—C18—C17	176.5 (4)	C19—C20—C21—C22	0.3 (12)
C2—C1—S1—C7	−104.9 (5)	C20—C19—C24—C23	−1.1 (10)
C2—C1—S1—C13	6.0 (6)	C20—C21—C22—C23	1.0 (12)
C2—C1—C6—C5	0.6 (9)	C21—C22—C23—C24	−2.4 (11)
C2—C3—C4—C5	−1.4 (10)	C22—C23—C24—C19	2.4 (10)
C3—C4—C5—C6	2.7 (10)	C24—C19—C20—C21	−0.3 (10)
C4—C5—C6—C1	−2.2 (10)	C25—S2—C19—C20	84.4 (6)
C6—C1—S1—C7	81.5 (5)	C25—S2—C19—C24	−93.9 (5)
C6—C1—S1—C13	−167.7 (5)	C25—S2—C31—C32	−19.6 (6)
C6—C1—C2—C3	0.6 (9)	C25—S2—C31—C36	159.5 (5)
C7—S1—C13—C14	7.8 (5)	C25—C26—C27—C28	−1.7 (9)
C7—S1—C13—C18	−167.2 (4)	C26—C25—C30—C29	−2.0 (8)
C7—C8—C9—C10	−0.1 (8)	C26—C27—C28—C29	1.8 (10)
C8—C7—C12—C11	−1.6 (8)	C27—C28—C29—C30	−2.1 (10)
C8—C9—C10—C11	−1.0 (9)	C28—C29—C30—C25	2.0 (8)
C9—C10—C11—C12	0.7 (9)	C30—C25—C26—C27	1.8 (9)
C10—C11—C12—C7	0.5 (9)	C31—S2—C19—C20	−25.7 (6)
C12—C7—C8—C9	1.4 (8)	C31—S2—C19—C24	155.9 (5)
C13—S1—C7—C8	−78.3 (5)	C31—S2—C25—C26	78.4 (5)
C13—S1—C7—C12	99.5 (4)	C31—S2—C25—C30	−100.2 (4)
C13—C14—C15—C16	−1.0 (8)	C31—C32—C33—C34	0.3 (10)
C14—C13—C18—C17	1.5 (8)	C32—C31—C36—C35	0.1 (10)
C14—C15—C16—C17	0.4 (9)	C32—C33—C34—C35	0.4 (10)
C15—C16—C17—C18	1.2 (9)	C33—C34—C35—C36	−0.9 (11)
C16—C17—C18—C13	−2.1 (8)	C34—C35—C36—C31	0.7 (11)
C18—C13—C14—C15	0.1 (8)	C36—C31—C32—C33	−0.5 (10)

### Triphenylsulfonium hexafluorophosphate (III) Crystal data

**Table:**

C_18_H_15_S^+^·PF_6_^−^	*F*(000) = 832
*M**_r_* = 408.33	*D*_x_ = 1.562 Mg m^−^^3^
Monoclinic, *P*2_1_/*n*	Cu *K*α radiation, λ = 1.54184 Å
*a* = 8.4524 (2) Å	Cell parameters from 4889 reflections
*b* = 18.1483 (5) Å	θ = 4.6–69.4°
*c* = 11.4344 (3) Å	µ = 3.10 mm^−^^1^
β = 98.251 (2)°	*T* = 100 K
*V* = 1735.84 (8) Å^3^	Irregular, clear colourless
*Z* = 4	0.27 × 0.18 × 0.09 mm

### Triphenylsulfonium hexafluorophosphate (III) Data collection

**Table:**

XtaLAB Synergy, Single source at home/near, HyPix3000 diffractometer	2782 reflections with *I* > 2σ(*I*)
Detector resolution: 10.0000 pixels mm^-1^	*R*_int_ = 0.038
ω scans	θ_max_ = 69.9°, θ_min_ = 4.6°
Absorption correction: multi-scan (CrysAlisPro; Rigaku OD, 2023)	*h* = −10→8
*T*_min_ = 0.225, *T*_max_ = 1.000	*k* = −21→22
8136 measured reflections	*l* = −12→13
3235 independent reflections	

### Triphenylsulfonium hexafluorophosphate (III) Refinement

**Table:**

Refinement on *F*^2^	0 restraints
Least-squares matrix: full	Hydrogen site location: inferred from neighbouring sites
*R*[*F*^2^ > 2σ(*F*^2^)] = 0.057	Only H-atom displacement parameters refined
*wR*(*F*^2^) = 0.158	*w* = 1/[σ^2^(*F*_o_^2^) + (0.0797*P*)^2^ + 2.0739*P*] where *P* = (*F*_o_^2^ + 2*F*_c_^2^)/3
*S* = 1.11	(Δ/σ)_max_ < 0.001
3235 reflections	Δρ_max_ = 1.03 e Å^−^^3^
250 parameters	Δρ_min_ = −0.76 e Å^−^^3^

### Triphenylsulfonium hexafluorophosphate (III) Special details

**Table:**

Geometry. All esds (except the esd in the dihedral angle between two l.s. planes) are estimated using the full covariance matrix. The cell esds are taken into account individually in the estimation of esds in distances, angles and torsion angles; correlations between esds in cell parameters are only used when they are defined by crystal symmetry. An approximate (isotropic) treatment of cell esds is used for estimating esds involving l.s. planes.

### Triphenylsulfonium hexafluorophosphate (III) Fractional atomic coordinates and isotropic or equivalent isotropic displacement parameters (Å^2^)

**Table:**

	*x*	*y*	*z*	*U*_iso_*/*U*_eq_	
S1	0.47256 (8)	0.22774 (4)	0.32198 (6)	0.0242 (2)	
P1	0.32535 (9)	0.08138 (4)	0.75173 (7)	0.0276 (2)	
F6	0.4296 (3)	0.15360 (12)	0.7797 (2)	0.0555 (7)	
F3	0.2176 (3)	0.00957 (13)	0.7247 (2)	0.0521 (6)	
F5	0.4752 (3)	0.04073 (15)	0.7153 (3)	0.0659 (8)	
F1	0.2725 (3)	0.10587 (17)	0.6204 (2)	0.0770 (10)	
F4	0.3778 (3)	0.05372 (19)	0.8831 (2)	0.0740 (9)	
F2	0.1740 (3)	0.11993 (18)	0.7935 (3)	0.0848 (11)	
C5	0.1043 (4)	0.08294 (17)	0.2990 (3)	0.0286 (7)	
H5	0.028270	0.065529	0.346123	0.032 (9)*	
C7	0.6285 (3)	0.17795 (16)	0.4110 (3)	0.0237 (6)	
C4	0.1048 (4)	0.05475 (17)	0.1864 (3)	0.0285 (6)	
H4	0.029907	0.017782	0.157169	0.034 (9)*	
C1	0.3235 (3)	0.16042 (16)	0.2728 (3)	0.0234 (6)	
C12	0.7692 (3)	0.21722 (17)	0.4441 (3)	0.0278 (7)	
H12	0.780691	0.266254	0.417475	0.034 (10)*	
C14	0.4035 (4)	0.26121 (16)	0.5460 (3)	0.0271 (6)	
H14	0.461917	0.218357	0.573505	0.037 (10)*	
C2	0.3247 (4)	0.13351 (17)	0.1587 (3)	0.0283 (6)	
H2	0.399730	0.151332	0.111099	0.026 (8)*	
C6	0.2139 (3)	0.13641 (17)	0.3432 (3)	0.0262 (6)	
H6	0.213792	0.156095	0.420153	0.037 (10)*	
C13	0.3877 (3)	0.28156 (16)	0.4282 (3)	0.0260 (6)	
C11	0.8916 (4)	0.18278 (18)	0.5168 (3)	0.0308 (7)	
H11	0.988554	0.208555	0.541217	0.043 (11)*	
C3	0.2137 (4)	0.08007 (17)	0.1165 (3)	0.0298 (7)	
H3	0.212555	0.060797	0.039141	0.035 (9)*	
C15	0.3320 (4)	0.30500 (18)	0.6228 (3)	0.0322 (7)	
H15	0.340487	0.292018	0.703904	0.031 (9)*	
C10	0.8746 (4)	0.11082 (18)	0.5548 (3)	0.0325 (7)	
H10	0.959295	0.087819	0.605437	0.038 (10)*	
C18	0.3044 (4)	0.34418 (18)	0.3850 (3)	0.0330 (7)	
H18	0.296378	0.357282	0.303882	0.066 (14)*	
C8	0.6083 (4)	0.10622 (17)	0.4460 (3)	0.0316 (7)	
H8	0.511436	0.080494	0.421077	0.048 (11)*	
C17	0.2333 (4)	0.38705 (17)	0.4633 (3)	0.0359 (8)	
H17	0.174288	0.429746	0.435789	0.044 (11)*	
C9	0.7337 (4)	0.07260 (19)	0.5188 (3)	0.0363 (8)	
H9	0.722888	0.023202	0.543915	0.054 (12)*	
C16	0.2482 (4)	0.36763 (18)	0.5817 (3)	0.0365 (8)	
H16	0.200411	0.397574	0.635155	0.037 (10)*	

### Triphenylsulfonium hexafluorophosphate (III) Atomic displacement parameters (Å^2^)

**Table:**

	*U*^11^	*U*^22^	*U*^33^	*U*^12^	*U*^13^	*U*^23^
S1	0.0253 (4)	0.0234 (4)	0.0240 (4)	−0.0026 (3)	0.0040 (3)	0.0032 (3)
P1	0.0275 (4)	0.0288 (4)	0.0262 (4)	−0.0007 (3)	0.0033 (3)	0.0007 (3)
F6	0.0520 (13)	0.0311 (11)	0.0732 (16)	−0.0071 (9)	−0.0258 (11)	0.0013 (10)
F3	0.0421 (11)	0.0504 (13)	0.0629 (15)	−0.0198 (10)	0.0049 (10)	0.0100 (11)
F5	0.0385 (12)	0.0649 (16)	0.098 (2)	−0.0059 (11)	0.0243 (13)	−0.0380 (15)
F1	0.0793 (18)	0.098 (2)	0.0434 (14)	−0.0533 (16)	−0.0255 (13)	0.0351 (14)
F4	0.0425 (13)	0.138 (3)	0.0390 (13)	−0.0271 (15)	−0.0031 (10)	0.0302 (15)
F2	0.0351 (12)	0.101 (2)	0.113 (2)	0.0200 (13)	−0.0067 (13)	−0.0619 (19)
C5	0.0249 (14)	0.0285 (15)	0.0328 (17)	−0.0018 (12)	0.0058 (12)	0.0014 (12)
C7	0.0235 (13)	0.0265 (15)	0.0218 (14)	0.0011 (11)	0.0054 (11)	−0.0012 (11)
C4	0.0272 (14)	0.0235 (14)	0.0334 (17)	0.0002 (12)	−0.0006 (12)	−0.0031 (12)
C1	0.0232 (13)	0.0238 (14)	0.0222 (14)	−0.0001 (11)	0.0003 (11)	0.0022 (11)
C12	0.0285 (15)	0.0262 (15)	0.0296 (16)	−0.0036 (12)	0.0074 (13)	−0.0051 (12)
C14	0.0303 (15)	0.0189 (14)	0.0327 (17)	−0.0024 (11)	0.0067 (13)	0.0012 (12)
C2	0.0299 (15)	0.0290 (15)	0.0265 (15)	0.0037 (12)	0.0062 (12)	0.0035 (12)
C6	0.0271 (14)	0.0296 (15)	0.0215 (14)	−0.0005 (12)	0.0017 (11)	0.0013 (12)
C13	0.0241 (14)	0.0217 (14)	0.0321 (17)	−0.0036 (11)	0.0038 (12)	−0.0010 (12)
C11	0.0253 (14)	0.0358 (17)	0.0311 (17)	−0.0015 (13)	0.0038 (12)	−0.0090 (13)
C3	0.0348 (16)	0.0290 (16)	0.0255 (16)	0.0030 (13)	0.0037 (13)	−0.0044 (12)
C15	0.0320 (16)	0.0307 (16)	0.0353 (18)	−0.0070 (13)	0.0095 (13)	−0.0046 (13)
C10	0.0297 (15)	0.0364 (18)	0.0301 (17)	0.0074 (13)	0.0002 (13)	−0.0030 (13)
C18	0.0273 (15)	0.0270 (16)	0.0428 (19)	−0.0017 (12)	−0.0017 (13)	0.0040 (13)
C8	0.0264 (15)	0.0277 (16)	0.0400 (18)	−0.0018 (12)	0.0028 (13)	0.0033 (13)
C17	0.0241 (15)	0.0224 (15)	0.059 (2)	0.0014 (12)	−0.0017 (14)	−0.0027 (14)
C9	0.0330 (16)	0.0304 (17)	0.044 (2)	0.0030 (13)	−0.0004 (14)	0.0060 (14)
C16	0.0251 (15)	0.0286 (16)	0.057 (2)	−0.0053 (12)	0.0102 (15)	−0.0151 (15)

### Triphenylsulfonium hexafluorophosphate (III) Geometric parameters (Å, º)

**Table:**

S1—C7	1.790 (3)	C14—C13	1.385 (4)
S1—C1	1.787 (3)	C14—C15	1.385 (4)
S1—C13	1.787 (3)	C2—H2	0.9500
P1—F6	1.586 (2)	C2—C3	1.387 (4)
P1—F3	1.594 (2)	C6—H6	0.9500
P1—F5	1.572 (2)	C13—C18	1.390 (4)
P1—F1	1.569 (2)	C11—H11	0.9500
P1—F4	1.585 (2)	C11—C10	1.390 (5)
P1—F2	1.590 (2)	C3—H3	0.9500
C5—H5	0.9500	C15—H15	0.9500
C5—C4	1.385 (4)	C15—C16	1.385 (5)
C5—C6	1.386 (4)	C10—H10	0.9500
C7—C12	1.392 (4)	C10—C9	1.389 (5)
C7—C8	1.380 (4)	C18—H18	0.9500
C4—H4	0.9500	C18—C17	1.387 (5)
C4—C3	1.382 (4)	C8—H8	0.9500
C1—C2	1.394 (4)	C8—C9	1.391 (5)
C1—C6	1.382 (4)	C17—H17	0.9500
C12—H12	0.9500	C17—C16	1.388 (5)
C12—C11	1.380 (4)	C9—H9	0.9500
C14—H14	0.9500	C16—H16	0.9500
			
C1—S1—C7	105.20 (13)	C1—C2—H2	120.8
C1—S1—C13	104.70 (13)	C3—C2—C1	118.4 (3)
C13—S1—C7	102.96 (14)	C3—C2—H2	120.8
F6—P1—F3	178.82 (14)	C5—C6—H6	120.7
F6—P1—F2	91.35 (15)	C1—C6—C5	118.5 (3)
F5—P1—F6	89.77 (13)	C1—C6—H6	120.7
F5—P1—F3	91.40 (13)	C14—C13—S1	121.5 (2)
F5—P1—F4	88.63 (16)	C14—C13—C18	122.5 (3)
F5—P1—F2	177.39 (19)	C18—C13—S1	116.0 (3)
F1—P1—F6	91.86 (13)	C12—C11—H11	119.6
F1—P1—F3	88.28 (13)	C12—C11—C10	120.8 (3)
F1—P1—F5	90.49 (18)	C10—C11—H11	119.6
F1—P1—F4	178.00 (18)	C4—C3—C2	120.3 (3)
F1—P1—F2	91.84 (19)	C4—C3—H3	119.8
F4—P1—F6	89.93 (14)	C2—C3—H3	119.9
F4—P1—F3	89.95 (14)	C14—C15—H15	119.8
F4—P1—F2	89.01 (17)	C16—C15—C14	120.3 (3)
F2—P1—F3	87.48 (14)	C16—C15—H15	119.8
C4—C5—H5	119.8	C11—C10—H10	120.0
C4—C5—C6	120.4 (3)	C9—C10—C11	119.9 (3)
C6—C5—H5	119.8	C9—C10—H10	120.0
C12—C7—S1	115.3 (2)	C13—C18—H18	120.9
C8—C7—S1	122.0 (2)	C17—C18—C13	118.3 (3)
C8—C7—C12	122.7 (3)	C17—C18—H18	120.9
C5—C4—H4	119.8	C7—C8—H8	120.9
C3—C4—C5	120.4 (3)	C7—C8—C9	118.2 (3)
C3—C4—H4	119.8	C9—C8—H8	120.9
C2—C1—S1	115.7 (2)	C18—C17—H17	120.0
C6—C1—S1	122.2 (2)	C18—C17—C16	120.0 (3)
C6—C1—C2	122.0 (3)	C16—C17—H17	120.0
C7—C12—H12	121.0	C10—C9—C8	120.4 (3)
C11—C12—C7	118.0 (3)	C10—C9—H9	119.8
C11—C12—H12	121.0	C8—C9—H9	119.8
C13—C14—H14	120.9	C15—C16—C17	120.6 (3)
C13—C14—C15	118.2 (3)	C15—C16—H16	119.7
C15—C14—H14	120.9	C17—C16—H16	119.7
			
S1—C7—C12—C11	177.4 (2)	C12—C7—C8—C9	0.9 (5)
S1—C7—C8—C9	−177.6 (3)	C12—C11—C10—C9	0.5 (5)
S1—C1—C2—C3	178.8 (2)	C14—C13—C18—C17	−0.8 (4)
S1—C1—C6—C5	−178.7 (2)	C14—C15—C16—C17	0.6 (5)
S1—C13—C18—C17	178.7 (2)	C2—C1—C6—C5	1.2 (4)
C5—C4—C3—C2	0.6 (5)	C6—C5—C4—C3	−0.6 (5)
C7—S1—C1—C2	−99.1 (2)	C6—C1—C2—C3	−1.1 (4)
C7—S1—C1—C6	80.8 (3)	C13—S1—C7—C12	−80.2 (2)
C7—S1—C13—C14	−22.8 (3)	C13—S1—C7—C8	98.4 (3)
C7—S1—C13—C18	157.7 (2)	C13—S1—C1—C2	152.7 (2)
C7—C12—C11—C10	0.5 (4)	C13—S1—C1—C6	−27.3 (3)
C7—C8—C9—C10	0.2 (5)	C13—C14—C15—C16	−0.5 (4)
C4—C5—C6—C1	−0.3 (4)	C13—C18—C17—C16	0.9 (4)
C1—S1—C7—C12	170.4 (2)	C11—C10—C9—C8	−0.9 (5)
C1—S1—C7—C8	−11.0 (3)	C15—C14—C13—S1	−178.9 (2)
C1—S1—C13—C14	87.0 (3)	C15—C14—C13—C18	0.6 (4)
C1—S1—C13—C18	−92.5 (2)	C18—C17—C16—C15	−0.9 (5)
C1—C2—C3—C4	0.2 (4)	C8—C7—C12—C11	−1.3 (4)
